# A pathogenic deletion in Forkhead Box L1 (FOXL1) identifies the first otosclerosis (OTSC) gene

**DOI:** 10.1007/s00439-021-02381-1

**Published:** 2021-10-11

**Authors:** Nelly Abdelfatah, Ahmed A. Mostafa, Curtis R. French, Lance P. Doucette, Cindy Penney, Matthew B. Lucas, Anne Griffin, Valerie Booth, Christopher Rowley, Jessica E. Besaw, Lisbeth Tranebjærg, Nanna Dahl Rendtorff, Kathy A. Hodgkinson, Leichelle A. Little, Sumit Agrawal, Lorne Parnes, Tony Batten, Susan Moore, Pingzhao Hu, Justin A. Pater, Jim Houston, Dante Galutira, Tammy Benteau, Courtney MacDonald, Danielle French, Darren D. O’Rielly, Susan G. Stanton, Terry-Lynn Young

**Affiliations:** 1 Faculty of Medicine, Memorial University, St. John’s, NL Canada; 2 Faculty of Health Sciences, National Centre for Audiology and School of Communication Sciences and Disorders, Western University, London, ON Canada; 3 Faculty of Science, Memorial University, St. John’s, NL Canada; 4 Department of Chemistry, University of Toronto, Toronto, ON Canada; 5 The Kennedy Centre, Department of Clinical Genetics, University Hospital, Rigshospitalet, Copenhagen, Denmark; 6 Institute of Clinical Medicine, University of Copenhagen, Copenhagen, Denmark; 7 Department of Otolaryngology, Head and Neck Surgery, London Health Sciences Centre, University Hospital, Western University, London, ON Canada; 8 ENT Consultants, St. John’s, NL Canada; 9 Eastern Health, St. John’s, NL Canada; 10 Department of Biochemistry and Medical Genetics, University of Manitoba, Winnipeg, MB Canada

## Abstract

Otosclerosis is a bone disorder of the otic capsule and common form of late-onset hearing impairment. Considered a complex disease, little is known about its pathogenesis. Over the past 20 years, ten autosomal dominant loci (*OTSC1-10*) have been mapped but no genes identified. Herein, we map a new *OTSC* locus to a 9.96 Mb region within the FOX gene cluster on 16q24.1 and identify a 15 bp coding deletion in Forkhead Box L1 co-segregating with otosclerosis in a Caucasian family. Pre-operative phenotype ranges from moderate to severe hearing loss to profound sensorineural loss requiring a cochlear implant. Mutant *FOXL1* is both transcribed and translated and correctly locates to the cell nucleus. However, the deletion of 5 residues in the C-terminus of mutant FOXL1 causes a complete loss of transcriptional activity due to loss of secondary (alpha helix) structure. *FOXL1* (rs764026385) was identified in a second unrelated case on a shared background. We conclude that *FOXL1* (rs764026385) is pathogenic and causes autosomal dominant otosclerosis and propose a key inhibitory role for wildtype Foxl1 in bone remodelling in the otic capsule. New insights into the molecular pathology of otosclerosis from this study provide molecular targets for non-invasive therapeutic interventions.

**Supplementary Information:**

The online version contains supplementary material available at 10.1007/s00439-021-02381-1.

## Introduction

Otosclerosis is a primary bone disorder of abnormal bone resorption and deposition in the otic capsule (bony labyrinth), and a common form of conductive hearing loss (HL). Although both environmental and genetic risk factors have been identified, pathogenesis of otosclerosis is unknown. Bone is a dynamic tissue that is continually remodelled, regulated by coupling signals between osteoclast and osteoblast cells involving interactions between a variety of factors including cytokines, chemokines, hormones, and biochemical stimuli. Why skeletal bone remodelling is required for health is not fully understood; however, its imbalance leads to disease, such as osteoporosis and inflammatory arthritis. The otic capsule is the rigid bony outer wall of the inner ear which protects the membranous labyrinth (endolymph-filled ducts) in its perilymph-filled cavities including the vestibule, semicircular canals, and cochlea. It is also unique in that it undergoes little remodelling after maturation compared to skeletal bones (0.13 vs. 10% yearly) (Frisch et al. 2000). The otic capsule retains small remnants of embryonic tissue (globuli interossei) containing cartilage and quiescent osteoclast and osteoblasts. In the early stages of otosclerosis, it becomes highly vascularized with activated macrophages (osteoclast progenitors) causing foci of reabsorption of endochondral bone and deposition of new dense bone by osteoblasts (Babcock and Liu 2018). Invasion of these osteosclerotic foci into the stapediovestibular joint immobilizes the stapes resulting in conductive HL (Nager 1969). The key molecular triggers in the otic capsule activating remodelling and the onset of otosclerosis remain elusive (Babcock and Liu 2018).

Clinical otosclerosis has a preponderance for Caucasians of northern European descent; first recognised as a common cause of HL in the 1800s (Ealy and Smith 2010), it is prevalent in 1/330 whites, 1/3300 blacks and 1/33,000 Asians (Thys and Van Camp 2009). Patients typically present with conductive HL which often progresses to mixed loss (cochlear otosclerosis); purely sensorineural hearing loss (SNHL) is rare. Onset is in the second, third or fourth decade and fortunately, the conductive component of otosclerosis is often successfully managed with a combination of stapes surgery and hearing aids (Cureoglu et al. 2006). In rare cases, a profound sensorineural deficit develops across all frequencies and requires cochlear implantation and electrical stimulation of the auditory nerve to restore hearing (Cureoglu et al. 2010). Histological otosclerosis refers to the observation that 4.5–12.5% of adult Caucasians show signs of otosclerosis post-mortem when temporal bones are examined. A definitive diagnosis of clinical otosclerosis requires visualization during surgery (Declau et al. 2001).

Although the majority of otosclerosis cases are considered sporadic, multiplex families have been used to map ten autosomal dominant (*OTSC*) loci to large genomic intervals: ***OTSC1*** (Indian; 15q25-qter; 14.5 Mb) (Tomek et al. 1998), ***OTSC2*** (Belgian; 7q34-q36, 16 Mb) (Van Den Bogaert et al. 2001), ***OTSC3*** (Cypriot; 6p22.3-p21.3, 17.4 Mb) (Chen et al. 2002), ***OTSC4*** (Israeli; 16q22.1-q23.1, 10 Mb) (Brownstein et al. 2006), ***OTSC5*** (Dutch; 3q22-q24, 15.5 Mb) (Van Den Bogaert et al. 2004), ***OTSC6*** (unpublished), ***OTSC7*** (Greek; 6q13-q16.1, 13.47 Mb) (Thys et al. 2007), ***OTSC8*** (Tunisian; 9p13.1-q21.11, 34.16 Mb) (Bel Hadj Ali et al. 2008), ***OTSC9*** (unpublished) and ***OTSC10*** (Dutch; 1q41-q44, 26.1 Mb) (Schrauwen et al. 2011) between 1998 and 2010. Although individual risk contributions are small, case–control association studies have identified susceptibility variants in genes involved in bone remodelling *COL1A1* (McKenna et al. 1998, 2004)*, TGFB1* (Thys and Van Camp 2009), *BMP2* and *BMP4 (*Schrauwen et al. 2008*)* and synaptic plasticity (*RELN*) (Schrauwen et al. 2009). Exploration of positional candidate genes in *OTSC2* patients suggests a potential role for the T cell receptor-beta gene in the dysregulated development of T-cells (Schrauwen et al. 2010). More recently, rare variants in *SERPINF1* (Ziff et al. 2016) in familial otosclerosis showed promise but failed to validate in a larger case and family series (Valgaeren et al. 2019). Despite the 20-year lapse since the mapping of *OTSC1*, the *OTSC* genes remain refractory to discovery due to the rarity of monogenic families, diagnostic challenges, and reduced penetrance. Herein, we map a new *OTSC* locus within the *FOX* gene cluster on 16q24.1 and identify *FOXL1* as the first *OTSC* gene.

## Materials and methods

### Discovery cohort-multiplex family from Newfoundland and Labrador (NL)

We have an ongoing recruitment drive for otosclerosis families from otolaryngology clinics in NL. One of the largest is Caucasian of English extraction segregating autosomal dominant otosclerosis of varied clinical presentation among seven surgically confirmed cases (Table 1). Onset of HL ranges from mid-teens to early twenties. In NL cases, PIDs II-6, II-9, II-11 and II-14, the abnormal bone remodelling that characterizes clinical otosclerosis primarily affected middle ear function, causing immobilization of the ossicular chain and a significant conductive hearing loss component, with minimal impact on the cochlea and sensorineural hearing sensitivity.

**Table 1:** Hearing phenotypes pre- and post-stapedectomy in confirmed otosclerosis cases

Subject (PID)	Onset (decade)	Hearing Loss diagnosis (L = Left; R = Right)			Surgical intervention/Clinical outcome		
		Ear	Type	Degree	Procedure	Age in years	Functional outcome
II-6 NL proband	3rd (early)	R	Mixed	Severe	Stapedectomy	52	Successful^b^
		L	Mixed	Moderately severe	Stapedectomy	52	Successful^b^
II-9	3rd (early)	R	Conductive	Severe	Stapedectomy	47	Partial success^c^
		L	Conductive or mixed	Unknown	Stapedectomy	22	successful^b^
II-11	3rd (early)	R	Sensorineural	Mild-severe	None	–	–
		L	Mixed	Moderate-severe	Stapedectomy	44	Successful^b^
II-14	3rd (early)	R	Conductive or mixed	Unknown	Stapedectomy	37	Successful^b^
		L	Conductive or mixed	Unknown	Stapedectomy	52	Partial success^c^
II-2	3rd	R	Conductive	Severe^a^	Stapedectomy	36	Unsuccessful^d^
		L	Mixed	Severe	Middle ear implant; cochlear implant	75, 76	Unsuccessful^d^, Successful^b^
II-3	2nd	R	Sensorineural	Profound	Stapedectomy; cochlear Implant	60, 61	Unsuccessful^d^, Successful^b^
		L	Sensorineural	Profound	None	–	–
III-2	2nd	R	Conductive^a^	Unknown^a^	Tympanomastoidectomy (for cholesteatoma) and Stapes mobilization	17, 18	Successful^b^, partial success^c^
		L	Conductive	Mild-moderate	None	–	–
IV-3 ON proband	3rd	R	Mixed	Profound	Stapedectomy	62	Successful^b^
		L	Sensorineural	Moderate low frequency	None	–	–

^a^Based on physician’s report

^b^Near complete resolution of conductive loss post stapedectomy; significant improvement of functional hearing post implant

^c^Hearing improved but significant conductive loss remains unresolved (> 20 dB air–bone gap at 2 or more frequencies)

^d^Either no hearing improvement or deteriorated hearing post-operatively

**Fig. 1:**
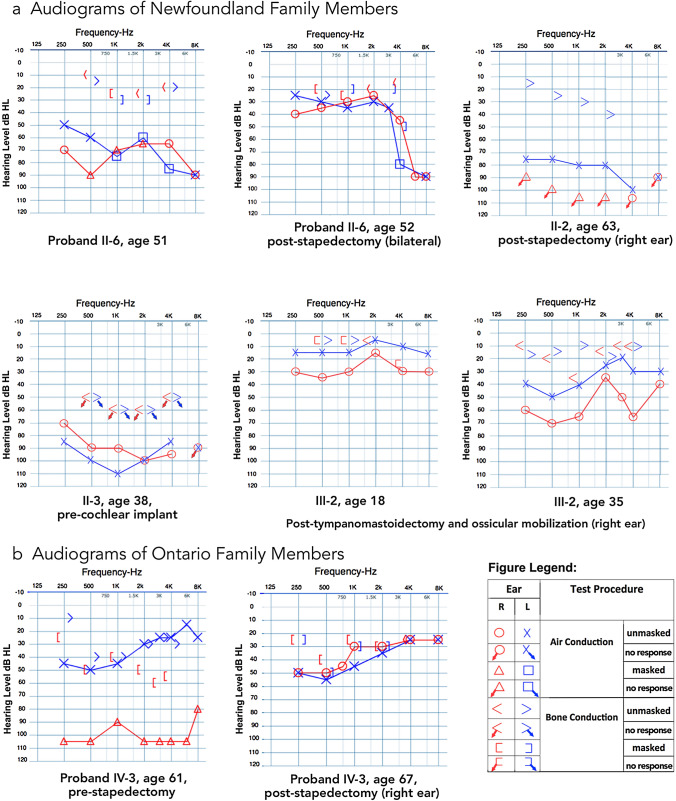
Selected audiograms of otosclerosis cases pre- and post-stapedectomy. **a** The NL proband (PID II-6) at age 51 showing extensive bilateral air-bone gaps, largely resolved by age 52 after bilateral stapedectomies. PID II-2 at age 63 presents with no measurable hearing after unsuccessful stapedectomy (R), and severe mixed loss (L). PID II-3 at age 38 presents with profound HL and no measurable bone conduction thresholds (bilateral). PID III-2 at age 18 shows normal hearing (L) and residual conductive loss following tympano-mastoidectomy and stapes re-mobilization (R), however, subsequent re-mobilization of right ear ossicles is not sustained and by age 35, conductive HL has progressed bilaterally. **b** The Ontario case at age 63 shows profound mixed HL (R) and moderate low-frequency sensorineural loss (L). By age 67 and following stapedectomy (R), air-bone gaps are largely resolved, and some bone conduction thresholds are improved (R) and bilateral HL is essentially symmetrical

The proband (PID II-6) at 25 years had bilateral conductive HL that progressed by age 51 to mixed asymmetric loss (R severe; L moderate-severe) with air-bone gaps averaging 50 dB and profound loss bilaterally at 8000 Hz (Fig. 1a, Table 1). Bilateral stapedectomies were clinically successful, with functional hearing significantly restored at 52 years and air-bone gaps resolved apart from slight residual conductive loss at 500 and 4000 Hz (Fig. 1a). High-frequency thresholds show minimal improvement post-operatively, consistent with possible cochlear otosclerosis and/or presbycusis.

**Fig. 2:**
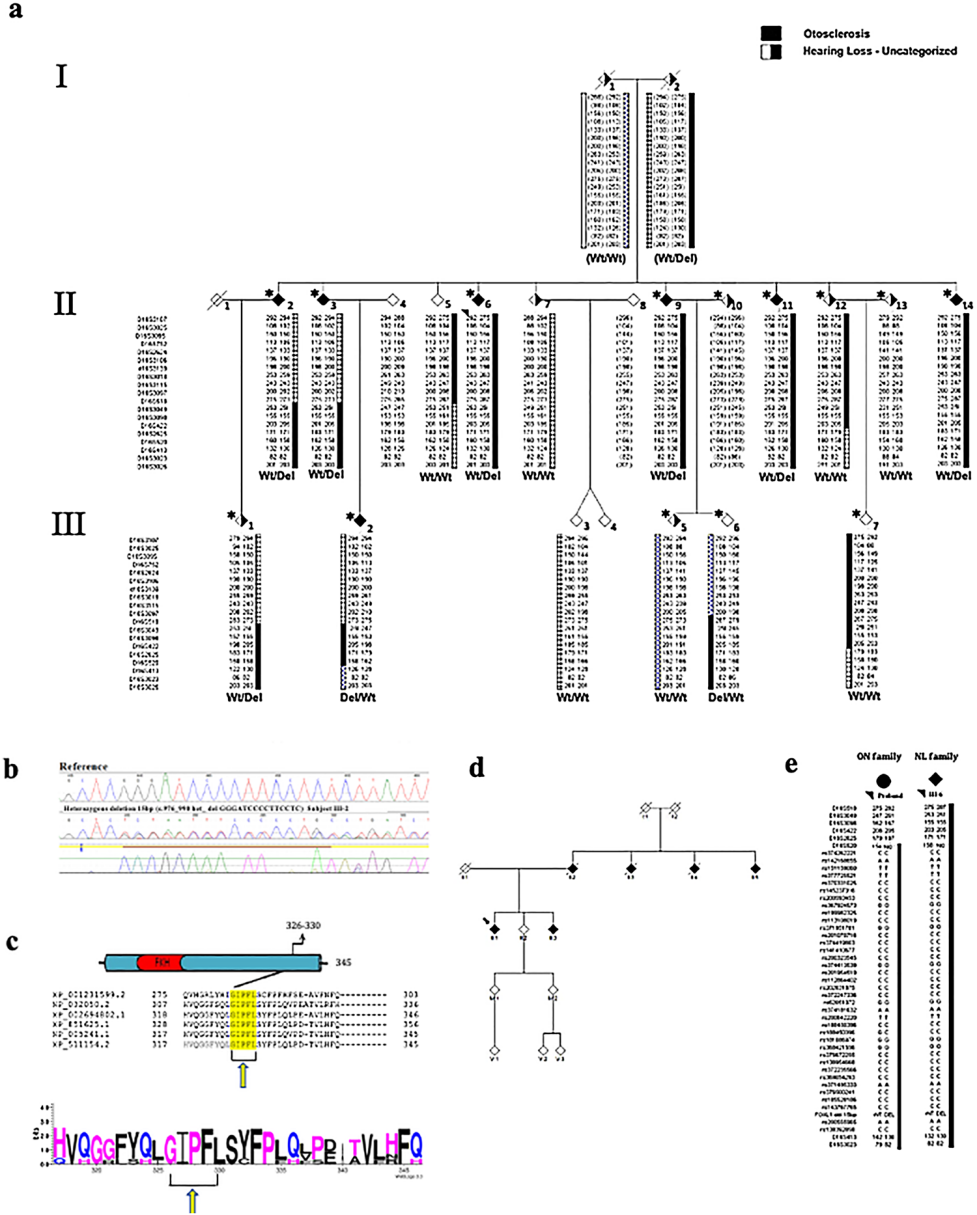
Co-segregation of a 15 bp In-Frame Deletion in Forkhead Box L1 (*FOXL1*, rs764026385) with Otosclerosis and Alignment of Orthologs. **a** Partial (core) pedigree from Newfoundland and Labrador (NL) with seven confirmed otosclerosis cases (filled symbols) used to map a new *OTSC* locus within the FOX gene cluster on chromosome 16q24.1. Asterisk (*) above symbols denote available audiograms. Recombination events to the disease haplotype (black) in siblings PIDs II-2, II-3 and II-5 positioned the causal gene qter of marker D16S518; a recombination in PID III-2 positioned the causal gene pter of D16S413. Taken together, these events refined the disease interval to a 9.96 Mb region on 16q24*.* Mutation status [Wt = wildtype; Del = *FOXL1*(rs764026385)] is shown and sex is masked (diamond symbol) to protect privacy. **b** Electropherogram of subject PID III-2 heterozygous for *FOXL1* (rs764026385). **c** Schematic of mutant Foxl1 (FKH = Fork head domain) with Weblogo display of aa conservation and the five missing residues (yellow) in the C-terminus of Foxl1; species [*G. gallus; XP_001231599.2, M. musculus; NP_032050.2, B. taurus;XP_002694802.1, C. lupus; XP_851625.1, H. sapiens; NP_005241.1, P.troglodytes; XP_511154.2*]*.* **d** Pedigree of Ontario otosclerosis case from the validation cohort identified as heterozygous for *FOXL1* (rs764026385). **e** Comparison of disease haplotype (black) within the critical disease interval (*D16S520*-*D16S413)* reveals haplotype sharing between the NL proband and the Ontario case, both are heterozygous for *FOXL1* (rs764026385)

The proband's parents (PIDs I-1, I-2, Fig. 2a) both have HL and several siblings (PID II-9, II-11, II-14) had a similar clinical course and successful post-stapedectomy resolution of conductive loss in one or both ears (Table 1). Although sibling PID II-2 had a similar early progression of conductive loss, stapedectomy (R) at age 36 was unsuccessful, resulting in profound loss with no response to stimuli. Amplification with a conventional hearing aid was used until no longer effective for severe mixed loss (L) (Table 1; Fig. 1a). By age 75, PID II-2 exhibited a more severe mixed loss and received a middle ear implant at age 75 which was unsuccessful. Subsequent cochlear implantation at age 76 provided substantial functional improvement. Sibling PID II-3 had HL by mid-teens and wore hearing aids by age 18. Cochlear otosclerosis was the primary cause of hearing loss, with no reliable responses to bone conduction stimuli recorded on repeated tests from age 33–38, consistent with the medical report of profound SNHL bilaterally at age 38, and inadequate benefit from hearing aids (Fig. 1a; Table 1). At 60 years of age, CT (computerized tomography) imaging confirmed “prominent” otosclerosis in PID II-3. Stapedectomy (R) was attempted without success. Cochlear implantation at age 61 significantly improved hearing function (self-report).

In the third generation, PID III-2 experienced an onset of conductive HL (R) in mid-teens, and was followed by surgical exploration at age 17, which identified stapes fixation and a cholesteatoma which precluded stapedectomy. The report of a follow-up tympano-mastoidectomy to remove the cholesteatoma notes that the ossicular chain was left intact and re-mobilized; however, complete resolution of the air-bone gap was not achieved (Fig. 1a, PID III-2, age 18) and subsequent ossicular re-mobilization achieved only temporary improvement. By age 35, the conductive loss had advanced to moderately severe (R) and moderate low-frequency conductive loss (L) resulting in diagnosis of otosclerosis at age 35 (Fig. 1a).

In all cases, patients were managed with conventional hearing aids, corrective stapes surgery and/or implanted middle and inner ear prostheses. Hearing thresholds improved dramatically post surgery, with near complete resolution of air-bone gaps, and noticeable improvement of bone conduction thresholds (Fig. 1B, age 67) consistent with previous reports (Sperling et al. 2013; Vijayendra and Parikh 2011). Genealogical investigations yielded a multigenerational Caucasian family with autosomal dominant otosclerosis.

### Clinical criteria for linkage analysis on the NL family

For linkage analyses (NL family only), we used conservative clinical criteria to assign affection status: affected were family members with otosclerosis confirmed by surgery; unaffected were blood relatives ≥ 60 years of age with normal (bilateral) hearing thresholds: all remaining members were considered "unknown status". High-resolution temporal bone CT was not available for most cases as it is not ordered routinely in Newfoundland and Labrador unless there is suspicion of SSCD (superior semicircular canal dehiscence) or for cochlear implant (CI). All available medical charts and audiological reports were reviewed, and patients assessed, when possible, to update audiograms and confirm middle ear status. Classification of HL was based on the pure-tone threshold average of 0.5, 1.0 and 2.0 kHz as defined by the American Speech and Hearing Association. A difference of > 10 dB HL between air and bone conduction sensitivity represented a significant conductive component associated with impaired sound transfer through the middle ear.

### Validation cohort

For validation purposes, we used unrelated otosclerosis cases recruited from Canada (*n* = 82), Finland (*n* = 35) and the Faroe Islands (*n* = 20). This study was approved by institutional review boards at Memorial University (#1.186), Western University (#103,679) and the Danish Research Ethical Committee (KF 01–234/02 and KF 01–108/03).

### Linkage to published OTSC loci and susceptibility genes

Genomic DNA was isolated from peripheral leukocytes according to a standard salting out procedure (Miller et al. 1988). Microsatellite markers were fluorescently labeled and amplified by PCR, run on ABI 3130xl or 3730 (Applied Biosystems) and analyzed with Gene Mapper v4.0. Pedigrees were drawn with Progeny (Progeny Genetics LLC) and haplotypes phased manually with the least number of recombination events. To test for linkage in the NL family, five affected members (PIDs II-2, II-3, II-6, II-9, III-2; otosclerosis confirmed by surgery) and two of unknown clinical status (PIDs III-5, III-6) (Fig. 2a) were genotyped for markers spanning each *OTSC* disease interval and bracketing three otosclerosis susceptibility genes (Supplementary Table 1). As well, a total of 17 relatives were genotyped with nine extra markers (*D16S518, D16S3049, D16S3098, D16S422, D16S2625, D16S520, D16S413, D16S3023, D16S3026*) mapping qter of the *OTSC4* disease interval. Two-point parametric linkage analyses (MLINK ver 5.1) were run for three markers per locus (assuming autosomal dominant inheritance, 99% penetrance and a gene frequency of 0.00). LOD scores were calculated at recombination fractions 0.000 to 0.5000. The proband was also sequenced for rare otosclerosis-associated variants in *SERPINF1* (Ziff et al. 2016).

### Sequencing, variant filtering and validation

Positional candidate genes (UCSC Genome Browser and NCBI build 36.) were selected for sequencing based on function and/or gene expression. Oligonucleotide primers were designed (Primer 3) to amplify the longest isoform and to include all intron/exon boundaries and UTRs. Samples were Sanger sequenced on an ABI 3130xl/3730. For first-pass variant filtering, we used three affected (PID II-3, II-9, III-2) and an unaffected spouse (PID II-4) (Fig. 2A). Variants either absent in affected samples or present in the unaffected spouse were removed from further study, as were variants with a MAF > 2% using dbSNP and 1000 genomes. We used multiple in silico tools (SIFT, PolyPhen-2, PANTHER, Human Splicing Finder) and ClustalW for aa (amino acid) conservation, to evaluate the potential functional consequences of filtered variants.

For completeness, we next ran whole-exome sequencing (WES) to screen all positional candidate genes. We selected five affected (PIDs II-2, II-6, II-9, II-11, II-14) and two senior (55, 60 yrs. old) population controls with normal hearing thresholds for first-pass variant filtering. WES was outsourced to the Genome Centre (McGill University, QC, Canada) including library preparation (TrueSeq Prep Kit). Samples were run on the Illumina Hiseq 2000, generating 50–150 million 100 bp paired end reads. Reads > 32 bp long were aligned to the 1000 genome reference using Burrows–Wheeler Aligner (BWA) and merged with Picard software (Broad Institute). Where multiple base mismatches and false positive variant calls were recorded, insertions and deletions were realigned using GATK software (DePristo et al. 2011; Van der Auwera et al. 2013). The percentage of aligned region coverage was detected using the Genome Centre's in-house database. The regions were identified as high coverage (> 400), low coverage (< 50), low mean mapq MQ (< 20) and no data. Variants either absent in affected(s) or present in normal hearing control(s) were removed from further study. The remaining rare (MAF < 1%) variants residing within linked regions (with a minimum of 20X coverage) were analysed with multiple in silico tools [samtools mpileup algorithm (Li et al. 2009), SnpSift (Cingolani et al. 2012), SnpEff (Cingolani et al. 2012), SIFT, PolyPhen-2 and PANTHER] including ClustalW to determine aa conservation. Variants of interest were validated by cascade sequencing in the NL family. Heterozygous variants co-segregating with otosclerosis in an autosomal dominant pattern were tested in the unrelated otosclerosis cases. Positive hits were genotyped and examined for potential allele sharing with the disease haplotype identified in the NL family.

### Computer modelling of 2D structure of mutant Foxl1 C-terminus

To assess the effect of the missing residues on FOXL1 structure, simulations were performed with the NAMD software (version 2.12) (Khajeh et al. 2020). The structure of the most C-terminal 69 residues of wildtype (FOXL1_CTERM_) and the deletion mutant (FOXL1_MUT_) were deduced by molecular dynamics simulations. Initial structures were generated in an extended state using the Protein in Atomistic details coupled with Coarse-grained Environment (PACE) model (Han and Schulten 2012). The protein was solvated in a cubic simulation cell with a side length of 90 Å Langevin thermostat with a damping coefficient of 10 ps^−1^ was employed to maintain temperature. All non-bonded interactions were shifted to zero between distances of 9 Å and 12 Å. The time-step for all simulations was set to 5 fs. This system was minimized, and an 8.5 ns equilibration molecular dynamics simulation was performed. Replica exchange molecular dynamics (REMD) (Zhou 2007) was then employed to sample the folded configurational space of these proteins. For both the mutant and wild-type proteins, 1 µs long simulations were performed with 32 replicas with temperatures ranging from 300 to 500 K. The dominant structure in the 300 K replica was determined by clustering analysis based on the root mean squared distance (RMSD) of all protein atoms. Clustering analysis was performed in VMD 1.9.1 program using a 3 Å RMSD criteria.

### Experimental measurements of 3D structure of mutant Foxl1 C-terminus

To validate modelling of the 3D structure, FOXL1_MUT_ and FOXL1_CTERM_ were produced recombinantly in *E. coli,* purified by nickel affinity chromatography followed by size-exclusion chromatography. Circular dichroism measurements were carried out on a Jasco J-810 spectropolarimeter (Jasco Inc.) in the far ultraviolet range (190–260 nm) with a 0.5 mm quartz cuvette at RT (average of 20 scans).

### Cell culture and RNA extractions

RNA was extracted from transformed B cell lymphocytes from both affected and unaffected individuals (controls) using Trizol-based methods (Thermo-fisher, Cat. #15,596,026). Osteoblast (hFOB 1.19) and HEK293A cell lines (ATCC) were maintained as adherent cells in Iscove’s Modified Dulbecco’s Medium (IMDM) F/12 (Life Technologies) supplemented with 10% heat inactivated FBS, 2 mM L-glutamine and antibiotic–antimycotic (100 units/ml penicillin G sodium, 100 µg/ml streptomycin sulfate, and 0.25 µg/ml amphotericin B as Fungizone®). hFOB 1.19 cells were maintained under the same culture conditions, but without phenol red, and G418 was added to a final concentration of 0.3 mg/ml. Lymphoblastoid cells were maintained in RPMI medium (Life Technologies). Total RNA was extracted using Trizol Reagent and treated with Ambion® TURBO™ DNase (Thermo Fisher). RNA was evaluated and quantified using a 2100 Bioanalyzer (Agilent) for samples with a RIN > 8.5 and cDNA synthesized with the High-Capacity cDNA Reverse Transcription Kit (Thermo Fisher). For mRNA expression, we used RT-PCR and *FOXL1* Taqman primers (L primer, CCTCCCTACAGCTACATCGC; R primer, TGTCGTGGTAGAAGGGGAA; hybrid probe, GGTCACGCTCAACGGCATCTA). *GAPDH* (Hs03929097_g1; Thermo Fisher) was used as an endogenous control (quantified by ∆∆CT method and normalized using the Viia7™ system, Thermo Fisher).

### FOXL1 expression constructs

To investigate the effect of the *FOXL1* 15-bp deletion on function, we transfected osteoblast cell line (hFOB1.19) with *FOXL1-WT* and *FOXL1-MUT* expression plasmids. We purchased two expression vector constructs (pReceiver-M02 and pReceiver-M29, cat. no. EX-E0843-M02, EX-E0843-M29-GFP) from GeneCopoeia containing wild-type *FOXL1* (NM_005250) and generated two mutant constructs (*FOXL1* c.976_990del) by site directed mutagenesis (NOROCLONE biotech laboratories), using two empty plasmids as mock controls. Transfection conditions were optimized using Fugene HD (Roche) transfection reagent, diluting plasmid with Opti-MEM (Thermo Fisher) to 0.02 µg/µl; Fugene HD in the ratio of 7:2 (Fugene HD in µl: plasmid DNA in µg) for 20 min at RT and hFOB 1.19 cells were transfected with 100ul transfection mixture in a 6-well plate and incubated for 48 h at 34 °C.

### Western blot

To determine the effect of the *FOXL1* deletion on the quantity and location of Foxl1 protein, we used osteoblast cells transfected with the wildtype and mutant constructs and immunoblotting. *FOXL1* expression was determined using Anti-FOXL1 rabbit polyclonal IgG (ab83000, Abcam) (1 μg/ml). Validation of antibody specificity was determined by comparing FOXL1 transfected to un-transfected cell lines for the presence of FOXL1 protein band according to molecular weight. Titration of each antibody was performed and the concentration yielding the highest signal-to-noise ratio was used in subsequent experiments. Housekeeping proteins were detected with α tubulin (clone DM1A + DM1B, Abcam) (200 μg/ml) and anti-nuclear matrix protein p84 antibody (clone 5E10, Abcam) (1 µg/ml). Horse Radish Peroxidase (HRP)-conjugated affiniPure F(ab)_2_ fragment goat anti-mouse (GAM) IgG, Fc specific and HRP-conjugated affiniPure F(ab)_2_ fragment goat anti-rabbit (GAR) IgG, and Fc specific antibodies (1:10,000 dilution) were obtained (Jackson Immunoresearch Labs Inc.). Protein lysates were obtained using the Nuclear Extract Kit (ActiveMotif) and protein concentration determined with the Bradford protein assay kit (BioRad). Samples were reduced using 0.2 M mercaptoethanol, 10 ug of protein per lane and subjected to 8% SDS PAGE. Following electrophoresis, proteins were transferred onto nitrocellulose and blocked with 5% milk powder in TBS-Tween (0.15 M NaCl, 0.05 Tris pH 7.4, 0.05% Tween 20). Primary antibodies were incubated at 4 °C overnight and HRP-conjugated secondary antibodies were used to detect antibody binding. Signals were amplified using a chemiluminescence detection agent (Millipore). Immunoreactivity was visualized by scanning densitometry (Image Quant LAS 4000) and quantified (Image GE software) (GE Healthcare).

### Fluorescence microscopy

To compare the localization of both wildtype and mutant Foxl1 protein, transfected HFOB1.19 cells were visualized with a Carl Zeiss AxioObserver A.1 microscope with standard fluorescence and brightfield/darkfield settings at X5 0,25 or X20 0,50 NA objectives. Images were captured using a Zeiss AxioCam MRM3 camera with Zeiss AxioVision 4.8 software. GFP-transfected cells were harvested by trypsin, followed by fixation in 1.0% paraformaldehyde (Sigma), and analysis of 5000–10,000 cells using a FACS Calibur flow cytometer (Becton–Dickinson).

### Luciferase reporter assay

To determine if the removal of 5 C-terminus residues in FOXL1 alters its ability to activate downstream transcription, we used a luciferase reporter assay. The reporter construct contained two copies of the *FOXL1* Consensus-binding sequence [TATACATAAACAAGAA] (Pierrou et al. 1994) cloned into pGL3 (Promega) upstream of the *thymidine kinase* minimal promoter and the luciferase open reading frame (*Photinus*). 1.0 μg of constructs bearing the wildtype, or 15 base-pair mutant ORFs, or an empty expression vector containing no *FOXL1* sequence, was co-transfected into HEK293 cells with 250 ng of *FOXL1* luciferase reporter and 10 ng of constitutively active (*Renilla*) luciferase in 24-well dishes. Luciferase activity was measured using the dual Luciferase Assay kit (Promega) and six wells for each treatment, repeated three times. Ratios of *Photinus* and *Renilla* luciferase were calculated, and wildtype and mutant readings were compared to those from the empty expression vector (which was standardized to 1). Data are graphed as mean fold change ± SD. A Fluoroskan Ascent (Labsystems) was used for all readings. The p value was calculated using a Student’s *t* test comparing the mean ratio (Photinus/Renilla) luciferase activity between wildtype and mutant Foxl1 transfections.

## Results

### Otosclerosis is Not Linked to Published OTSC Loci or Susceptibility Genes

In the NL family, recapitulating haplotypes mapping to published *OTSC* loci [*OTSC1* (15q25-q26), *OTSC2* (7q34q36), *OTSC3* (6p21.3-p22.3), *OTSC4* (16q22.1-q23.1)*, OTSC5* (3q22-24), *OTSC7* (6q13-16.1), *OTSC8* (9p13.1-9q2*)*, *OTSC10* (1q41-q44)] and susceptibility genes [*COL1A1*, *COL1A2*, *NOG*] failed to identify shared haplotypes among surgically confirmed cases (Abdelfatah 2014). Furthermore, significantly negative two-point LOD scores (< 2.0) supported linkage exclusion across all published *OTSC* loci (Supplementary Table 2). The proband also screened negative for rare variants recently identified in the *SERPINF1* gene (Ziff et al. 2016).

### New OTSC Locus Maps to 16q24 in NL family

Serendipitously, we noted allele sharing among affected members of the NL family for markers near the *OTSC4* qter boundary (Brownstein et al. 2006). Increased recruiting efforts and extensive genotyping on all available relatives mapped a disease-associated haplotype 12 Mb downstream of *OTSC4*. Two-point linkage analysis between surgically confirmed otosclerosis and 16q24 markers yielded positive LOD scores. Maximum LOD scores (LOD = 1.63; theta = 0) were achieved for two adjacent markers (D16S422 and D16S2625) suggestive of linkage (Supplementary Table 2). Using both affected and unaffected members, we identified key recombination events to the disease haplotype and defined a new *OTSC* locus spanning 9.96 Mb on 16q24 (Fig. 2a).

### Rare coding in-frame deletion in FOXL1 identified in the NL family

Sanger sequencing for 12 positional candidates (*PLCG2, IRF8, SCL38A8, ZDHHC7, SLC7A5, HSD17B2, COTL1, FOXF1, FOXL1, FOXC1, CA5A*, *OSGIN*) spanning the new *OTSC* locus on 16q24 yielded 92 variants. Of these, 91 variants were filtered out because they were either absent in affected samples, present in the unaffected spouse, had a MAF > 2% or predicted using in silico tools to be benign. The remaining variant, an in-frame deletion of 15bps in the transcription factor *FOXL1* (NM_005250.3: c.976_990del), was both absent in 116 ethnically matched controls and co-segregated with otosclerosis in the NL family (Fig. 2a, b). Subsequently, WES of all positional candidate genes uncovered two additional variants (*PKD1L2*; c.749 A > G and c.658 C > T); however, these variants failed segregation analysis. No other SNPs/deletions were detected. *FOXL1* has a single exon encoding a 345 aa transcription factor and resides in a gene cluster on 16q24.1 with *FOXC2* and *FOXF1*. The 15 bp in-frame deletion predicted the removal of residues 326–330 (GIPFL) from the C-terminus of *Foxl1* (Fig. 2c)*.* The *FOXL1* (rs764026385) variant is reported to have a global frequency of 0.211% [highest in European (non-Finnish) subpopulation at 0.247%; rarest in the African subpopulation at 0.060%; gnomAD] but has not been reported in Clinvar or ClinGen. A single report in Varsome classified this variant as a VUS according to ACMG criteria (Richards et al. 2015).

### Positive FOXL1 hit in validation cohort

Targeted screening of *FOXL1* (rs764026385) in the unrelated otosclerosis series identified a case (PID III-1, Fig. 2d) from the Canadian province of Ontario with allele sharing, suggesting a common ancestor (Fig. 2e). The Canadian case was heterozygous for the same deletion identified in the NL family and phenotypically had profound mixed loss (R) and moderate low-frequency SNHL (L) (Fig. 1b, Table 1). Acoustic immittance testing revealed absent acoustic middle ear muscle reflexes despite normal middle ear compliance, a hallmark feature of otosclerosis. Follow-up interview with the proband revealed a family history of vertical transmission of hearing loss, consistent with autosomal dominant inheritance.

### Structural consequences of deletion FOXL1 p.(Gly326_Leu330del)

**Fig. 3:**
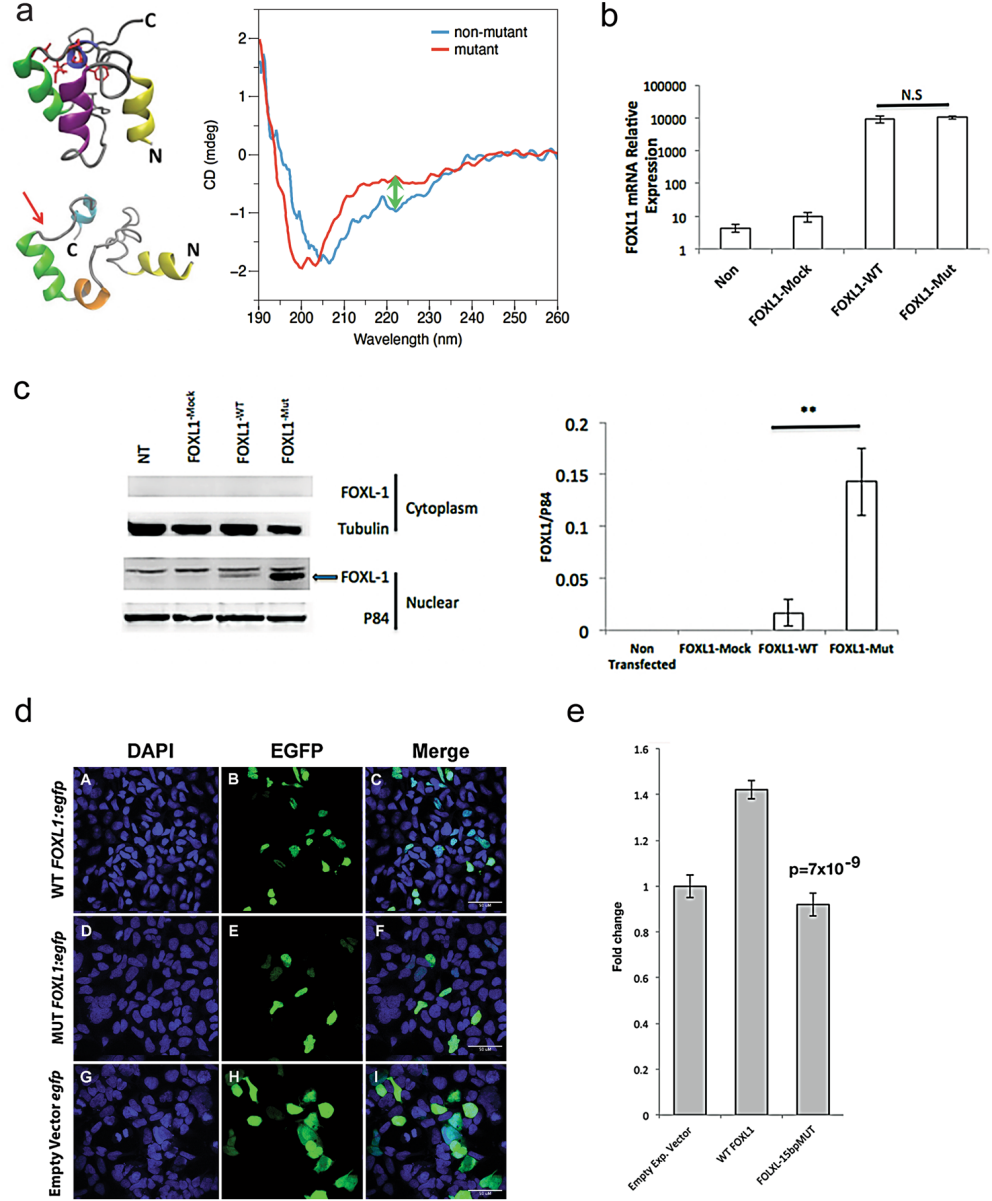
Structural and functional investigation of the 15-bp *FOXL1* in-frame deletion. **a** Computational modeling displaying wildtype Foxl1 (top left) and misfolded Foxl1 (bottom left), the result of disrupting the hydrophobic core (arrow) producing a randomly coiled structure. Secondary structure (right) experimentally probed by circular dichroism (CD) in both wildtype and mutant Foxl1 produced recombinantly in *E. coli*. Note that wildtype Foxl1 appears to have twice as much helix as the mutant (arrow), the C-terminus of mutant Foxl1 is mostly random coil (48% of the helicity of wildtype). **b** In osteoblasts (hFOB1.19), both FOXL1 (hFOB-FOXL1-Mut) and the wildtype (hFOB-FOXL1) constructs express RNA to a high level (Non = non-transfection control; FOXL1-Mock = empty vector control). Bar graph represents the mean *FOXL1* mRNA relative expression ± SD of three independent experiments. GAPDH was used as an endogenous control. **c** In osteoblasts (hFOB1.19), cytoplasmic and nuclear extract proteins were prepared and immunoblotted using a FOXL1 antibody (ab83000) using αTubulin (DM1A + DM1B) and P84 (5E10) as cytoplasmic and nuclear loading controls, respectively (left). Protein levels assessed by Western blotting show significantly increased (**p < 0.01) expression (right) of FOXL1 mutant (FOXL1-Mut) compared with FOXL1 wildtype (FOXL1-WT). FOXL1 levels were normalized to P84 and the average band intensity after normalization is presented in the bar graph. Error bars represent the ± SD of three independent experiments. **d** Expression of wildtype and mutant FOXL1:GFP fusion proteins in HEK293 cells. Transfection of constructs containing wildtype FOXL1;egfp fusion proteins demonstrates that wildtype FOXL1:egfp (panel B) localizes to the nucleus (panel A), as evidenced by their co-localization (panel C). Similarly, expression of mutant FOXL1:egfp fusion proteins (panel E) localizes to the nucleus (panel D) as evidenced by their colocalization (panel F). Expression of empty vector egfp [no FOXLQ ORF, (panel H)] localizes to both the nucleus (panel G) and cytoplasm, as evidenced by egfp detection within and beyond the DAPI stain (panel I). **e** In HEK293 cells, transfection of constructs containing the wild-type *FOXL1* ORF (WT FOXL1) increased transcription from the FOXL1 reporter by 42% over endogenous levels using a luciferase transcription assay. Transfection with the mutant ORF does not induce transcription from the reporter (FOXL1-15bpMUT), indicating a loss of transcriptional activity (*p* = 7 × 10^–9^). Bar graph represents pixel intensity ± SD of three independent experiments

The in-frame deletion of five residues in the C-terminal domain of *FOXL1* in otosclerosis patients occurs in the most ordered and evolutionary-conserved portion of FOXL1. Simulations showed that removal of residues GIPFL disrupted the hydrophobic core resulting in an increasing randomly coiled structure (Fig. 3a). The C-terminus of the wildtype and mutant FOXL1 was also produced recombinantly in *E. coli* and their secondary structure probed experimentally by circular dichroism (CD). As indicated by the difference in ellipticity at 222 nm, the wildtype structure has twice as much helix as the mutant, suggesting that the mutant and wild-type proteins have substantially different structures, the mutant being more random coil, while the wild type has a substantial amount of alpha helix (Fig. 3a). We estimate that C-terminus of the mutant FOXL1 has 48% of the helicity of wildtype FOXL1.

### Functional consequences of FOXL1 p.(Gly326_Leu330del)

As *FOXL1* RNA expression level was undetectable using transformed B-cell lymphocytes from subjects, cell lines were transfected with either FOXL1-^WT^ or FOXL1-^MUT^ expression plasmids to compare gene and protein expression. Osteoblast cell lines (hFOB1.19) transfected with either FOXL1-^WT^ or FOXL1-^MUT^ expression plasmids show a high level of RNA expression with RT-PCR Taqman assay (Fig. 3b). Transfection efficiency was identical in both wild-type and mutant *FOXL1* constructs. Immunoblotting revealed FOXL1 protein was significantly increased (**p < 0.01) in the mutant and expression was localized to the nucleus (Fig. 3c). Expression of wildtype and mutant FOXL1: GFP fusion proteins in HEK293 cells also indicates that wildtype FOXL1 localizes to the nucleus (Fig. 3d). To test the transcriptional activity of mutant *FOXL1*, using human HEK293 cells, we constructed a luciferase reporter with two copies of the *FOXL1* consensus binding sequence to drive expression from a minimal promoter (thymidine kinase). While transient transfection of the wildtype *FOXL1* constructs increased luciferase expression 42% over endogenous levels, no transcriptional activity (*p* = 7 × 10^–9^) above endogenous levels was observed in cells transfected with the *FOXL1* mutant (Fig. 3e). Taken together, we conclude that *FOXL1* (NM_005250.2: c.976_990del) is pathogenic according to the following hearing loss and indel specific ACMG criteria (PS3_Moderate, PM4, PP1_Strong, PP3, PP4) and the cause of autosomal dominant otosclerosis in this study (Oza et al. 2018; Richards et al. 2015).

## Discussion

We have mapped and identified the first *OTSC* gene, Forkhead Box L1, in a Caucasian family of English extraction. Using haplotype and linkage exclusion, we ruled out previously published *OTSC* loci and several associated genes and mapped a new *OTSC* locus within the FOX gene cluster on chromosome 16q24.1. Significant research resources were dedicated to family recruitment and clinical assessment, which turned out to be essential to mapping the new *OTSC* locus and identifying the causal gene by reducing the number of rare heterozygous variants that required functional follow-up. RNA and protein investigations reveal that *FOXL1* deletion variant is both transcribed and translated and correctly localizes to the cell nucleus. However, the missing residues in the C-terminus of mutant FOXL1 cause significant loss of helical structure, rendering the mutant transcription factor devoid of transcriptional activity suggesting haploinsufficiency. A second unrelated Caucasian case was heterozygous for *FOXL1* (rs764026385) and resides on the same disease haplotype as the NL family, suggesting a common ancestor and the likelihood that this *OTSC* variant was imported to North America from northern Europe. Genomics England reveals a MAF of 0.0033 (509/156,390 alleles: 503 heterozygous and 3 homozygous individuals). The fact that several cases of homozygosity of the *FOXL1* (rs764026385) have been identified is not inconsistent with non-lethal dominant variants relatively common in the population. We reported a case of homozygosity of a pathogenic *WFS1* variant causing autosomal dominant low-frequency hearing loss in a NL family (Young et al. 2001).

Abnormal bone remodelling that characterizes otosclerosis usually impairs middle ear function by limiting stapes mobility, with minimal impact on the cochlea during the early stage of the disease process. NL family members underwent stapes surgery to improve their conductive hearing loss, which was successful with exceptions. While the left ear postoperative outcome was positive for PID II-9, right ear stapedectomy at age 47 was complicated by facial nerve dehiscence causing friction against the piston prosthetic which compromised full recovery of hearing and no further intervention was attempted due to the risk of facial nerve paralysis. PID II-14 also had a successful right ear stapedectomy at age 37 and at age 52, left stapedectomy improved hearing at most frequencies but some hearing loss persisted at 1000 Hz for reasons unknown. PID II-2 had right stapedectomy at age 32, however, subsequent to this procedure, it became apparent that no residual hearing remained, consistent with possible perilymph fistula, a known risk of stapedectomy surgery. With disease progression, sensorineural hearing sensitivity can decline if otosclerosis involves the cochlea. This was the case for PID II-2, who developed a more severe mixed loss in the left ear by 75 years of age. A purely cochlear type of otosclerosis is a less common clinical presentation, and with a significant sensorineural hearing loss component, stapes surgery is of limited benefit. One family member, PID II-3 had predominantly cochlear otosclerosis. Despite this diagnosis, a right ear stapedectomy was performed (at age 60) in an attempt to reduce the conductive component and restore hearing thresholds sufficiently for hearing aid use. When this less invasive surgery was unsuccessful, the surgeon proceeded to right cochlear implantation (at age 61).

In skeletal bones, bone remodelling is determined by the interplay between receptor activator of nuclear factor (NF)-kB-ligand (RANKL), which binds to the receptor activator of NF-kB (RANK) on progenitors inducing osteoclastogenesis and osteoprotegerin (OPG), which prevents RANKL from binding to RANK. In the otic capsule, the same RANK, RANKL and OPG axis are at play, however, bone turnover is suppressed by the release of OPG into the perilymph by fibrocytes in the spiral ligament (cochlear lateral wall). High levels of OPG maintain this suppression in the membranous labyrinth of the inner ear. OPG binding to RANKL regulates both bone resorption and spiral ganglion degeneration, as studies in knockout Opg − / − mice reveal the development of conductive hearing pathology due to abnormal bone growth and SNHL caused by auditory nerve degeneration (Kao et al. 2013).

FOX proteins are a super family of transcription factors that play increasingly recognised roles in a diverse range of developmental processes, such as the establishment of the body axis and the development of tissues from all three germ layers. Specifically, FOX proteins lie at the junction of multiple signalling pathways and play crucial roles in regulating gene expression in cell metabolism, proliferation, differentiation and apoptosis (Lam et al. 2013). In the last decade, the identification of causal genes for rare, monogenic skeletal dysplasia has provided novel insights into the role and functioning of the Wingless and int-1 (WNT) signalling pathway. The result of discoveries in monogenic dysplasia, the WNT signalling pathway is known to be important in both skeletal development and bone homeostasis. In the otic capsule, *FOXL1* likely acts upon the globuli interossei containing cartilage and quiescent osteoclast and osteoblasts. Zebrafish studies show that *Foxl1* can have both transcriptional activation and repression functions and transcriptional repressor Foxl1 regulates central nervous system development by suppressing sonic hedgehog (shh) expression (Nakada et al. 2006).

In the NL family, PID III-2 was diagnosed with cholesteatoma at 17 years of age. β‐catenin (part of WNT pathway) expression is increased in cholesteatoma cells when compared with normal epithelium cells and we postulate that cholesteatoma, like otosclerosis, may be a result of mutation in *FOXL1*, given reported cases of familial cholesteatoma, including rare autosomal dominant families (Collins et al. 2020; Jennings et al. 2018). Industry focus on pharmacologically modulating the Wnt signalling pathway in cancer provides a potential treatment option for otosclerosis and possibly cholesteatoma as well (Zimmerli et al. 2017).

Strengths of this study included conservative "affected" status that avoided phenocopies. This is particularly important with phenotypes like HL that are common, as seen in the NL family where both pedigree founders had HL. It is also important to recognise that phenotypes change over time, and multiple distinct diagnosis may represent the changing face of a monogenetic disorder under study in a large family. A conservative definition of otosclerosis was especially critical during positional cloning as variant filtering with just one incorrect affected or unaffected case would hamper gene discovery. A comprehensive approach using traditional (linkage and haplotype analysis) in combination with new (NGS) technologies and significant resources to recruit and clinically assess blood relatives helped minimize the critical disease interval to the smallest among published *OTSC* loci. Going forward, it will be important to clinically follow two discordant NL family members (PIDs III-1, PID-III-6) to determine if they are pre-clinical or truly non-penetrant.

The genetic architecture of autosomal dominant otosclerosis includes at least 11 genes (*OTSC1-OTSC10* plus *FOXL1*) and likely many variants within each gene. Limitations of this study include a limited series of otosclerosis cases which precluded our ability to estimate the prevalence or penetrance of *FOXL1* otosclerosis in northern Europeans. Penetrance is defined as the portion of individuals in the general population with a particular variant that displays the trait. Of the 9 *FOXL1* deletion carriers identified in this study, 8 are members of the NL family used to identify the *FOXL1* gene and the other is the proband of a family from the validation cohort, so we are unable to estimate the penetrance of the *FOXL1* pathogenic variant with a limited number of biologically- related gene carriers. Uncorrectable ascertainment, where the most clinically affected families are recruited, is an essential design feature of gene discovery, but will likely overestimate the true penetrance of any gene. A much larger, global case series is required to evaluate the contribution of *FOXL1* to both Mendelian and sporadic otosclerosis cases. Interestingly, the *FOXL1* variant is most frequent in Europe and rarest in Africa, which may contribute to the low prevalence of otosclerosis in Africa. However, caution should be exercised regarding the perceived prevalence of otosclerosis (and other types of late-onset HL) in understudied populations, especially in non-European populations. Another limitation was the time to forge collaborations and successfully complete multiple functional studies, which added to the time from gene discovery to publication, a recognised challenge to the timely publication of new gene discoveries (Bamshad et al. 2019).

We call for renewed efforts to identify all *OTSC* genes, as determining the factors that trigger bone turnover in the inner ear remain elusive. The combination of positional cloning and NGS was successful in this study and so far has resolved 92% of the monogenic skeletal dysplasias (Huybrechts et al. 2020). Otosclerosis is considered to be 40% penetrant (Moumoulidis et al. 2007); however, the true penetrance of each *OTSC* mutation awaits their discovery and testing to identify gene carriers in the general population to circumvent ascertainment bias. Although developing animal models of *OTSC* genes will provide mechanistic insights into disease pathology, accurate diagnosis and therapeutic treatment of otosclerosis requires the identification of all the *OTSC* genes. As stated by Bamshad et al. "*the vast majority of variants of known function in the human genome underlie Mendelian conditions, and the study of natural genome variation manifest by Mendelian conditions still provides a time-efficient and cost-effective path to link genotype with human phenotype.*" (Bamshad et al. 2019). This study provides new insights into genes and pathways involved in osteosclerosis and renewed hope that therapeutic options for otosclerosis and perhaps other bone remodelling disorders are now within striking distance.

## Web resources

Amer Speech &Hearing Assoc, https://www.asha.org/practice-portal/clinical-topics/hearing-loss/

ATTC, https://www.atcc.org/

Burrows–Wheeler Aligner BWA, http://bio-bwa.sourceforge.net/

ClinGen, https://clinicalgenome.org/

ClinVar, https://www.ncbi.nlm.nih.gov/clinvar/

ClustalW, https://www.genome.jp/tools-bin/clustalw

dbSNP, https://www.ncbi.nlm.nih.gov/snp/

gnomAD, https://gnomad.broadinstitute.org/

Hereditary Hearing Loss homepage, http://hereditaryhearingloss.org/

Human Splicing Finder, www.umd.be/HSF3/

NCBI, ncbi.nlm.nih.gov/

OMIM, https://www.ncbi.nlm.nih.gov/omim

1000 genomes, https://www.internationalgenome.org/home

Primer3, https://bioinfo.ut.ee/primer3-0.4.0/

PANTHER, http://www.pantherdb.org/

PolyPhen-2, http://genetics.bwh.harvard.edu/pph2/

RefSeq, https://www.ncbi.nlm.nih.gov/refseq/

SIFT, https://sift.bii.a-star.edu.sg/

SNP database, http://www.ncbi.nlm.nih.gov/projects/SNP/

Varsome, http://varsome.com

Weblogo, https://weblogo.berkeley.edu/logo.cgi

UCSC Genome Browser, https://genome.ucsc.edu/

## Supplemental data

Supplemental Data include Table 1 (markers used for haplotyping), Table 2 (two-point linkage (LOD) scores) and Fig. 1 (audiograms of non-penetrant FOXL1 p.(Gly326_Leu330del) deletion carriers.

## Supplementary Information

Below is the link to the electronic supplementary material.

Supplementary file1 (DOCX 32902 KB)

## Data Availability

The datasets generated during and/or analysed during the current study are available from the corresponding author on reasonable request.
