# Supplementary material for: A pathogenic deletion in Forkhead Box L1 (FOXL1) identifies the first otosclerosis (OTSC) gene
Source: Hum Genet. 2021 Oct 11;141(3-4):965–79. doi: 10.1007/s00439-021-02381-1 (PMC9034980; doi:10.1007/s00439-021-02381-1)
Supplement: Supplementary file 1 — Supplementary file1 (DOCX 32902 KB) [file 439_2021_2381_MOESM1_ESM.docx]

# TITLE: A Pathogenic Deletion in Forkhead Box L1 (*FOXL1*) Identifies the First Otosclerosis (*OTSC*) Gene

Nelly Abdelfatah,^1^ Ahmed A. Mostafa,^1^ Curtis R. French,^1^ Lance P. Doucette,^1^ Cindy Penney,^1^ Matthew B. Lucas,^2^ Anne Griffin,^1^ Valerie Booth,^3^ Christopher Rowley,^3^ Jessica E. Besaw,^4^ Lisbeth Tranebjærg,^5,6^ Nanna Dahl Rendtorff,^5^ Kathy A. Hodgkinson,^1^ Leichelle A. Little,^2^ Sumit Agrawal,^7^ Lorne Parnes,^7^ Tony Batten,^8^ Susan Moore,^1^ Pingzhao Hu,^10^ Justin A. Pater,^1^ Jim Houston,^1^ Dante Galutira,^1^ Tammy Benteau,^1^ Courtney MacDonald,^1^  Danielle French,^1^  Darren D. O’Rielly,^1,9^ Susan G. Stanton,^2^**^†^** Terry-Lynn Young^1^**^†*^**

^1^Faculty of Medicine, Memorial University, St. John’s, NL, Canada; ^2^National Centre for Audiology & School of Communication Sciences and Disorders, Faculty of Health Sciences, Western University, London, ON, Canada; ^3^Faculty of Science, Memorial University; ^4^ Department of Chemistry, University of Toronto, ON, Canada. ^5^The Kennedy Centre, Department of Clinical Genetics, University Hospital, Rigshospitalet, Copenhagen, Denmark; ^6^Institute of Clinical Medicine, University of Copenhagen; ^7^Department of Otolaryngology, Head & Neck Surgery, London Health Sciences Centre, University Hospital, Western University, London, ON, Canada; ^8^ENT Consultants, St. John’s, NL; ^9^Eastern Health, St. John’s, NL; ^10^Department of Biochemistry and Medical Genetics, University of Manitoba, Winnipeg, MB, Canada.

†These authors contributed equally to this work.

*Correspondence: [tlyoung@mun.ca](mailto:tlyoung@mun.ca)

**Supplemental Table 1.** Microsatellite markers genotyped spanning the disease intervals of 8 published OTSC loci and 3 otosclerosis associated genes.

* Markers mapping adjacent to published markers that were uninformative.

| Locus | Location | Disease Interval  (Mb) | Microsatellite Markers (**Boundary Marker**) |
| --- | --- | --- | --- |
| *OTSC1* | 15q25-q26 | 14.5 | ****D15S127****, D15S652, D15S649, D15S1004, D15S157,* ***D15S657*** |
| *OTSC2* | 7q34-q36 | 16 | ***D7S495****, D7S684, D7S2202, D7S2513, D7S676, D7S1798, D7S2442,* ***D7S2426****, D7S1827* |
| *OTSC3* | 6p22.3-p21.3 | 17.4 | ***GAAT3A06****, D6S1660, D6S1545, D6S464, D6S273 D6S1568, D6S291, D6S1602,* ***D6S1680*** |
| *OTSC4* | 16q22.1q23.1 | 10 | ***D16S3107****, D16S3025, D16S3095, D16S752, D16S3106, D16S3139 D16S3018, D16S3115,* ***D16S3097*** |
| *OTSC5* | 3q22-p24 | 15.5 | ***D3S1292****, D3S3641, D3S1576, D3S3586, D3S3694, D3S1593, D3S3627,* ***D3S1744*** |
| *OTSC7* | 6q13-q16.1 | 13.47 | ****D6S467****, D6S280, D6S1596, D6S456, D6S1589, D6S460 D6S1652, D6S1595 D6S1613,*****D6S450*** |
| *OTSC8* | 9p13.1-9q21.11 | 34.16 | ***D9S970,*** *D9S1844, D9S1862, D9S1879, D9S166****, D9S1799*** |
| *OTSC10* | 1q41-q44 | 26.1 | ***D1S2621****, D1S439, D1S2800,* ***D1S2811*** |
| *COL1A1* | 17q21.33 | - | ***D17S797****, D17S1795, D17S941, D17S809,* ***D17S788*** |
| *COL1A2* | 7q21.3 | - | ***D7S644****, D7S657, D7S2430, D7S821,* ***D7S651*** |
| *NOG* | 17q22 | - | ***D17S790****, D17S1607, D17S1606, *****D17S1161*** |

**Supplementary Table 2.** Two-point LOD scores between otosclerosis and microsatellite markers spanning eight *OTSC* loci, three otosclerosis-associated genes and a new *OTSC* locus qter of *OTSC4* (Extended *OTSC4*).

| **Locus** | **Markers** | **Recombination fraction**  **0.000 0.100 0.200 0.300 0.400 0.500** |
| --- | --- | --- |
| *OTSC1* | D15S127  D15S649  D15S657 | -4.096936 -0.887153 -0.387585 -0.151425 -0.035454 0.000000  -4.096936 -0.264114 -0.108875 -0.048774 -0.014267 0.000000  -0.300990 -0.099513 -0.029093 -0.005594 -0.000348 0.000000 |
| *OTSC2* | D7S495  D7S1798  D7S1827 | 0.301029 0.212668 0.130215 0.062729 0.016819 0.000000  -4.096936 -0.887153 -0.387585 -0.151425 -0.035454 0.000000  -13.494847 -1.33082 -0.581401 -0.227145 -0.053183 0.000000 |
| *OTSC3* | GAAT3A06  D6S273  D6S1680 | -3.795907 -0.887043 -0.387574 -0.151424 -0.035454 0.000000  -3.795907 -0.887043 -0.387574 -0.151424 -0.035454 0.000000  -3.795907 -0.653891 -0.226226 -0.062029 -0.007427 0.000000 |
| *OTSC4* | D16S3107  D16S3106  D16S3097 | -4.342935 -0.766770 -0.300033 -0.086144 0.002510 0.000000  -4.342935 -0.636432 -0.200119 -0.038453 0.003295 0.000000  -4.342935 -0.598769 -0.218146 -0.063158 0.001094 0.000000 |
| Extended *OTSC4*  *(16q24)* | D16S422  D16S2625  D16S520 | 1.630082 1.269478 0.884142 0.488383 0.143531 0.000000  1.630082 1.287609 0.908845 0.502244 0.140761 0.000000  -0.176089 -0.176583 -0.153096 -0.092488 -0.028034 0.000000 |
| *OTSC5* | D3S3548  D3S1593  D3S1744 | 0.301025 0.210206 0.118188 0.044152 0.006429 0.000000  -4.096936 -0.755097 -0.299465 -0.104434 -0.021215 0.000000  -3.795907 -0.653881 -0.226224 -0.062028 -0.007427 0.000000 |
| *OTSC7* | D6S1619  D6S1595  D6S268 | -3.795906 -0.638142 -0.218710 -0.059726 -0.007201 0.000000  -4.096936 -0.264114 -0.108875 -0.048774 -0.014267 0.000000  -3.795906 -0.638142 -0.218710 -0.059726 -0.007201 0.000000 |
| *OTSC8* | D9S2148  D9S1862  D9S1799 | -4.045783 -0.199700 -0.047532 -0.007852 -0.000417 0.000000  -13.494847 -0.672523 -0.254094 -0.086982 -0.018424 0.000000  -0.300990 -0.099513 -0.029093 -0.005594 -0.000348 0.000000 |
| *OTSC10* | D1S2621  D1S2800  D1S2811 | -4.096936 -0.264114 -0.108875 -0.048774 -0.014267 0.000000  0.000000 0.000000 0.000000 0.000000 0.000000 0.000000  -4.096936 -0.264114 -0.108875 -0.048774 -0.014267 0.000000 |
| *COL1A1* | D17S797  D17S809  D17S788 | -0.300990 -0.248075 -0.151917 -0.069146 -0.017365 0.000000  -4.096936 -0.443629 -0.193798 -0.075713 -0.017727 0.000000  -4.096936 -0.443629 -0.193798 -0.075713 -0.017727 0.000000 |
| *COL1A2* | D7S644  D7S821  D7S651 | -3.795907 -0.013984 0.073263 0.053196 0.016338 0.000000  -3.795902 0.025915 0.139021 0.118035 0.047724 0.000000  0.602028 0.465356 0.318042 0.170249 0.049214 0.000000 |
| *NOG* | D17S790  D17S1606  D17S1161 | -4.698990 -0.691749 -0.345732 -0.144865 -0.035094 0.000000  -4.096936 -0.755097 -0.299465 -0.104434 -0.021215 0.000000  -4.096936 -0.755097 -0.299465 -0.104434 -0.021215 0.000000 |

**Supplementary Figure 1.** Audioprofiles of the two discordant (non-penetrant) *FOXL1* (rs764026385) carriers from the NL family. Audiogram of PID III-1 with unilateral hearing loss consistent with recreational noise exposure at age 27 (upper R panel) and with bilateral high frequency hearing loss at age 54 following occupational noise exposure (upper L panel). Audiograms of PID III-6 at ages 28 and 35 showing normal hearing thresholds (bottom L and R panels, respectively).
